# Towards a more accurate and reliable evaluation of machine learning protein–protein interaction prediction model performance in the presence of unavoidable dataset biases

**DOI:** 10.1515/jib-2024-0054

**Published:** 2025-04-02

**Authors:** Alba Nogueira-Rodríguez, Daniel Glez-Peña, Cristina P. Vieira, Jorge Vieira, Hugo López-Fernández

**Affiliations:** Instituto de Investigação e Inovação em Saúde (i3S), Universidade do Porto, Rua Alfredo Allen 208, 4200-135 Porto, Portugal; SING Research Group, Galicia Sur Health Research Institute (IIS Galicia Sur), SERGAS-UVIGO, 36213 Vigo, Spain; Instituto de Biologia Molecular e Celular (IBMC), Rua Alfredo Allen, 208, 4200-135 Porto, Portugal

**Keywords:** protein–protein interaction prediction, machine learning, biased datasets, model evaluation, sequence embedding

## Abstract

The characterization of protein-protein interactions (PPIs) is fundamental to understand cellular functions. Although machine learning methods in this task have historically reported prediction accuracies up to 95 %, including those only using raw protein sequences, it has been highlighted that this could be overestimated due to the use of random splits and metrics that do not take into account potential biases in the datasets. Here, we propose a per-protein utility metric, pp_MCC, able to show a drop in the performance in both random and unseen-protein splits scenarios. We tested ML models based on sequence embeddings. The pp_MCC metric evidences a reduced performance even in a random split, reaching levels similar to those shown by the raw MCC metric computed over an unseen protein split, and drops even further when the pp_MCC is used in an unseen protein split scenario. Thus, the metric is able to give a more realistic performance estimation while allowing to use random splits, which could be interesting for more protein-centric studies. Given the low adjusted performance obtained, there seems to be room for improvement when using only primary sequence information, suggesting the need of inclusion of complementary protein data, accompanied with the use of the pp_MCC metric.

## 1 Introduction

Protein-protein interactions (PPIs) control most biological activities in living organisms, making them fundamental to cellular functions [1]. PPIs can be classified based on their composition, affinity and life time/stability [2], 3]. According to composition, they can be homo- and hetero-oligomeric complexes, if PPIs occur between identical chains, or if PPIs take place among non-identical chains [4]. Regarding affinity, they can be classified into non-obligate and obligate complexes. These differ in affinity, and components (protomers, monomers) of non-obligate interactions can exist independently in contrast with obligate complexes where the constituents are unstable on their own. According to lifetime and stability, PPIs can be permanent interactions (interactions that occur for a long period of time, such as those of metabolic and regulatory networks) or transient (interactions happen under certain biological contexts, in which association/dissociation occurs quickly, such as those of signaling and secretory pathways [5], 6]. PPIs identification is essential for the characterization of protein functions and to the understanding of the molecular mechanisms in the cells [7]. Such information is also essential to the identification of disease mechanisms, which facilitate the therapeutic target identification and novel drug design [8], 9]. Innovative techniques, including low-throughput methods like nuclear magnetic resonance spectroscopy and high-throughput approaches such as Yeast two-hybrid (Y2H) and Affinity Purification coupled with Mass Spectrometry [10], have transformed the identification of protein-protein interactions (PPI) across various organisms. However, challenges remain due to the time and economic costs associated with studying the extensive proteome of living organisms and the potential number of interactions of interest. For this reason, computational prediction of PPI using machine learning (ML) algorithms is gaining increasing relevance [11]. These algorithms are used not only as complementary evidence to experimental methods but also to predict novel interactions that can then be experimentally addressed. ML algorithms can use protein sequences, protein structure, and co-fractionation mass-spectrometry data to extract relevant features to make predictions [12].

Among machine learning (ML) approaches, deep learning (DL), can automatically extract effective features from large-scale original datasets without prior knowledge [12]. Deep neural networks (DNNs) are capable of identifying long-range interactions in data, such as the amino acid residues used in protein interactions in a protein sequence in 1D space, and thus are used for task classification [12]. These methodologies use patterns within the protein sequence such as amino acid combinations, and the protein sequence that define folding motifs, from known interacting polypeptides, that can be used to predict PPIs based on similarity of protein sequences [12]. Examples of PPI prediction sequence-based prediction methods that use DNN for capturing features of primary sequences in 1D space (Table 1 in [12]) are DeePPI (Deep neural networks for Protein–Protein Interactions prediction) [13], DNN-PPI (deep neural network framework) [14], and PIPR (Protein–Protein Interaction Prediction Based on Siamese Residual) [15]. DeePPI extracts high-level discriminative features from sequence specificities of DNA and RNA binding proteins, DNN-PPI capture features in high-dimensional position-specific profiles of protein sequences such as semantic associations between amino acids, position-related sequence segments (motif), and their long- and short-term dependencies, features learned automatically from protein primary sequences, and PIPR leverages on robust local features and contextualized information, and incorporates deep residual recurrent neural network for capturing features of primary sequences and do not use spatial information of protein molecules, in the Siamese architecture.

The datasets used for PPI prediction based on protein sequence in 1D space methods are made of reference protein sequences and data on whether two proteins interact or not, that can be split into training and validation sets. It should be noted that in order to determine the real performance of ML methods, the training and validation datasets should be carefully analyzed to avoid potential biases and pitfalls. Data on interacting proteins is obtained from databases such as STRING (Search Tool for the Retrieval of Interacting Genes/Proteins, https://string-db.org/) which provides known and predicted protein-protein interactions and is regularly updated by the authors [16]. Another well-known database used both at a general level and filtering by organisms whose content focuses on interactions between experimentally verified proteins is DIP (Database of Interacting Proteins, https://dip.doe-mbi.ucla.edu/dip/Main.cgi). There are also specialized databases such as HPRD (Human Protein Reference Database) [17] focused on human PPIs. It is also crucial to have knowledge of non-interacting proteins, although there are few databases focused on collecting such data. One such database is Negatome [18], which gathers information on protein pairs known not to interact with each other from scientific literature and experimental data.

The prediction accuracy of these primary sequences in 1D space methods has been reported to be higher than 95 %, which is surprising since they do not take into consideration the 3D structure of protein complexes, binding pockets, domains, surface residues and binding affinities [10]. Nevertheless, recently, it has been suggested that data leakage (sequence similarities and node degree information) in training and test sets are associated with high prediction accuracy, and that when data leakage is avoided performance becomes random [19], 20]. In this context, we conducted a previous study on biases in two protein–protein interaction (PPI) datasets that may overestimate the performance of machine learning models with random splits such as cross-validation [21]. We used pre-trained language models (SeqVec and ProtBert) to extract embeddings from protein sequences, combining them through concatenation, addition and multiplication. We trained several machine learning algorithms and developed a baseline model (PPIIBM) to predict protein interactions. Our results revealed a significant bias in the data, which could inflate performance metrics by relying on prior protein positivity (see Section 3).

Here, in this follow-up study, we present a metric that aims at better estimating the real performance in biased PPI datasets. We analyze whether the proposed metric is sufficient to avoid overestimating the power of PPI prediction based on protein sequence in 1D space methods, in comparison to ensuring that validation splits are only conformed of interactions of unseen proteins. Additionally, we incorporate ESM-2, a new language model for processing protein sequences.

## 2 Related work

Dealing with raw protein sequences in machine learning methods, as in natural language processing, often requires first extracting the relevant features that capture the full sequence information and embed it into a fixed-length space. Recently, DL word embedding methods are also being ported to the protein language, i.e. amino acid sequences.

We could differentiate among three generations of word embeddings. In the first generation, word embeddings are always the same regardless of the surrounding words in the document. Word2Vec [22], through its Skip-Gram model [23], is one of the most representative.

In the second generation, word embeddings became context-sensitive, meaning that the same words will have a different embedding, depending on the surrounding words. Recurrent neural networks, such as LSTM (Long Short-Term Memory) networks, were one of the most popular approaches. In the case of protein sequences, SeqVec [24], is a representative example of this generation, where ELMo (Embeddings from Language Models), a type of LSTM, were trained over proteins in UniRef50 database. Authors highlight the effectiveness of ELMo to capture biophysical properties of the proteins from unlabeled datasets (UniRef50). They tested the usefulness of their 1,024-length embeddings for several downstream tasks, including protein-level tasks, such as subcellular location prediction. The 1,024-length protein-level embeddings were obtained by averaging amino acid embeddings across the entire sequence.

The third generation takes advantage of the more recent transformer architectures [25] to model protein sequence’s language. Transformers are more efficient than previous recurrent neural networks for training, and are able to handle wider context windows, which enables them to model natural language with an unprecedented success. From the same team as SeqVec, ProtTrans [26] trained Transformer-XL and XLNet and four auto-encoder models (BERT, Albert, Electra, T5) in Uniref50, Uniref100 and BFD100. As with SeqVec, 1,024 fixed-length embeddings seem to capture biophysical features and authors have used them as feature extractors for two per-protein downstream prediction tasks, including the subcellular location prediction and membrane versus water-soluble prediction by using a single feed forward layer. Authors found that bi-directional models (auto-encoders), such as BERT, outperformed SeqVec in protein-level predictions tasks.

Along these lines, Meta AI’s ESM (Evolutionary Scale Modeling; https://github.com/facebookresearch/esm) also emerged, a family of language models developed specifically for biology and bioinformatics, focusing on protein sequences. These transformer-based models are designed to understand and predict the structures and functions of proteins from their amino acid sequences. ESM-2 represents the latest generation of the ESM family, where there are different models that vary in size and dimensionality of the embeddings (320–5,120 fixed-length embedding). In this case, we focus on an embedding size similar to that of ProtBert, so we will work with a 1,280 fixed-length embedding [27].

Focusing on the PPI prediction task, Chen et al. [15] developed a model named PIPR that pre-trains the Skip-Gram model [23] on protein sequences to obtain amino acid embeddings of the two input proteins, that are then directly fed into an specific end-to-end DL model, based on Siamese Residual Recurrent Convolutional Neural Networks (R-RCNN), able to extract features for the input protein sequence pairs, designed for several prediction tasks, including binary PPI prediction.

Song et al. [28] proposed another end-to-end DL framework, known as TAGPPI, which employed SeqVec [24] for amino acid embeddings, that are then used to (i) extract sequence features by using TextCNN and (ii) structural features, by using Graph Attention Networks (GAT) over the AlphaFold DB contact map predictions (https://alphafold.ebi.ac.uk), respectively. After fusing both feature sets, a final feature vector for each protein is obtained, and finally concatenated to feed a MLP for PPI prediction.

Wu et al. [29] proposed a new DL architecture, named DL-PPI, that relies on Skip-Gram and InceptionV3 to get protein-level embeddings that are then fed to Graph Neural Networks (GNNs) for relationship-level features of each protein, followed by a self-attention layer to concentrate in the more relevant information of each protein and a final Neural Tensor Network (NTN) for PPI prediction.

Among others, all these studies included the Yeast dataset [30], widely used as a benchmark dataset in this area. Predictive performance results in general are very high (e.g. PIPR reached 97 % of balanced accuracy). However, a recent work from Bernett et al. [19] just reported that almost all PPI datasets used for evaluating such approaches are randomly split into train and test sets using cross-validation. This fact has been already pointed out by other studies that showed that this causes an inflation of prediction performance due to training data leakage [21], 31]. Bernett et al. trained and evaluated six sophisticated DL sequence-based PPI prediction methods, including PIPR, on seven well-known datasets, demonstrating that the high levels of accuracy reported in academic papers can be entirely attributed to the leakage of training data. More concretely, they report that methods seem to (i) exploit node degree information, since their performance is unaffected when the PPI graph is rewired while preserving node degree and (ii) sequence similarity, since all models fell to random-level performance when train/tests splits were done in such a way that minimizes the inter-split protein sequence similarity. Remarkably, Bernett et al. [19], 32] also provided a gold standard dataset for sequence-based PPI prediction that was specifically designed to prevent data leakage and minimize biases due to sequence similarity.

In this context, studies such as TUnA (Transformer-based Uncertainty Aware Model for PPI prediction) [32] appear with the aim of predicting protein-protein interactions with high accuracy and whose model evaluation is reliable. TUnA uses protein embeddings generated by ESM-2 to capture rich and complex representations of protein sequences, together with Transformer-based architecture to process these representations to capture intra-protein and inter-protein relationships and a Spectral-normalized Neural Gaussian Process (SNGP) in the final layer to provide uncertainty estimates in the predictions.

## 3 Materials and methods

### 3.1 Previous work

A systematic bias in two protein–protein interaction (PPI) datasets (Wei’s [33] and Yeast [30] dataset) that could lead to overestimation of ML model performance under classical random splits such as cross-validation has been previously investigated [21]. We employed a transfer learning method by preprocessing protein sequences using pretrained language models like SeqVec [24] and ProtBert [26] to extract features, calculating embeddings from protein sequences. These embeddings are combined through concatenation, addition, and multiplication to construct a final PPIs dataset. Concatenation appends the embeddings preserving all the input embeddings (vector AB), addition and multiplication of element-wise operations (vector A + B and vector A ⊙ B, respectively). Additionally, in the concatenation combination, we apply data augmentation by considering reverse pairings (vector “AB” and vector “BA”) for each interaction, aiming at enhancing model robustness. The results of each of these combinations were used to train machine learning algorithms such as k-nearest neighbors, logistic regression, random forests, and support vector machines to predict the interaction between proteins. Additionally, we built a Pair Prediction by Item Identification Baseline Model (PPIIBM) to perform a binary classification of pairs of items according to their relationship status. The model uses only the combination of concatenation and is based on learning the proportion of positive relationships for each element of the pair. PPIIBM is able to exploit a potential bias that arises when the same items appear in multiple pairs and when individual or both items show trends toward positive or negative relationships (see in Section 3.4 of [21]). In other words, datasets were unbalanced in terms of the positivity of each protein, that is, the ratio between positive and negative interactions per protein. Since this bias was more pronounced in the Wei dataset, where 94.4 % of proteins have only positive or negative interactions, the present work focuses only in the Yeast dataset, where, while a per-protein positivity unbalance still exist, is not as pronounced as in Wei dataset. Only 14.26 % of proteins have all positive or all negative interactions, but the bias is still present, since many proteins are skewed towards positive or negative relations. This bias could lead models to rely on the protein’s prior positivity to optimize accuracy during training. When evaluating the models, if they rely solely on the protein’s prior probability, it may result in inflated metrics. As this bias is exploited by the PPIIBM model, we achieved competitive metrics compared to other more complex models (see Section 4.2 in [21]), suggesting that these models might have an overestimated performance. To evaluate the effect of per-protein positivity imbalance on performance metrics, the need arises to evaluate models at the protein level.

Since no significant differences have been found with respect to SeqVec and ProtBert, we have decided for this study to focus on the latest generation models. Therefore, in the present study, we have focused on the new generation of transformers: ProtBert and ESM-2 model.

In the same sense, the combination of concatenation with data augmentation did not show significant improvements either, however, the computational cost was much higher, and therefore, we have decided not to continue with the data augmentation technique for this study.

### 3.2 ProtBert versus ESM-2 features

Advanced language models such as ESM (Evolutionary Scale Modeling), developed by Meta AI, and ProtBert, developed by ProtTrans, have emerged as powerful tools for protein sequence analysis. ESM is based on transformer architectures specifically tailored to capture evolutionary and structural relationships in protein sequences, training on extensive datasets such as Uniprot, BFD (Big Fantastic Database), MGnify and Pfam. On the other hand, ProtBert uses an adaptation of BERT focused on functional and structural prediction of proteins, trained mainly on data from UniRef100 and BFD. Both models are highly effective in predicting protein structure and function but, given the differences in approach, each model performs differently depending on the objective of the study [26], 27].

In this study, we use these language models to generate the protein embeddings as averages (per-residue embeddings are not used here) that are further used to train classical machine learning models. Both models are pretrained with protein information and inference is made to obtain the embedding of each protein with the last layer of the model. Table 1 shows the configurations for pre-training of the models used.

**Table 1:** Main characteristics of ESM-2 and ProtBert.

Model	ProtBert	ESM-2
Model name	prottrans_bert_bfd	esm2_t33_650M_UR50D
Number of parameters	420 M	650 M
Number of layers	30	33
Embedding dim	1,024	1,280
Number of attention heads	16	20
Training steps	880 K/200 K	500 K
Learning rate	0.002	0.0004
Weight decay	0.01	0.01
Dataset	BDF100 (2,122 million protein sequences)	UniRef50 v2021.04 (45 million protein sequences)
Download	https://huggingface.co/Rostlab/prot_bert_bfd	https://huggingface.co/facebook/esm2_t33_650M_UR50D

### 3.3 Experimental setup

In this study, the main focus was on using the Yeast dataset to train classical machine learning models, including k-Nearest Neighbors (KNN), logistic regression (LR), and random forest (RF), employing the embeddings generated by the ProtBert and ESM-2 models. This dataset comprises 2,497 distinct proteins. Of these, 76 proteins exhibit only positive interactions and 280 proteins exhibit only negative interactions. In contrast, 2,141 proteins have both positive and negative interactions. The total interactions consist of about 11,200 interactions showing an even global balance among positive and negative interactions. However, it is important to note that this global balance is not present at per-protein level, as it was previously commented (see Previous Work), where each protein could be biased towards positive or negative interactions.

The experiments were conducted in a computational environment with an AMD Ryzen ThreadRipper Pro 3955WX CPU at 3.9 GHz, 256 GB of RAM, and a total of seven Gigabyte RTX 3090 OC GPUs, each with 24 GB of VRAM. Each combination of model and embeddings was evaluated using a nested 5-fold cross-validation (CV) approach, in which each internal CV was aimed at tuning the model hyperparameters according to the parameter grids provided in Table 2. Then, each external CV evaluated the performance of the best hyperparameter configuration found by retraining the model using all training data and evaluating it on the test set.

**Table 2:** Parameter grids for the three ML models in the nested 5-fold CV.

Model	Parameter grid
KNN	‘n_neighbors’: [25, 75, 125]
Logistic regression	‘C’: [0.0001, 1, 10] ‘penalty’: [‘l1’, ‘l2’]
Random forest	‘n_estimators’: [100, 200] ‘min_samples_leaf’: [1, 10, 50] ‘max_samples’: [0.75, 1.0]

To explore the influence of unseen protein splits, the process was repeated twice, one run with the random interactions splits and another with unseen protein splits, maintaining a consistent number of iterations in both the internal and external CV as seen in Table 3. In the unseen protein splits case, random interaction splits were created initially and then, all training interactions involving any protein present in the corresponding validation partition were removed. Thus, the random interaction split was run with a 3-fold cross-validation, while the unseen protein split was run with 50-fold cross-validation. This methodology allows having a similar number of training interactions in each iteration and therefore comparing the results of the models under different data split conditions.

**Table 3:** Data split for training and validation of the model for execution in nested cross validation.

	Random splits		Unseen protein splits	
	No.	Training interactions per iteration	No.	Training interactions per iteration
Outer CV	3	±7,500	50	±7,700
Inner CV	3	±5,000	40	±5,200

In addition, a recently published gold standard dataset for sequence-based PPI prediction that was specifically designed to prevent data leakage and minimize biases due to sequence similarity [19], 34] was also analysed under the proposed framework. It contains 163,192 training interactions, 59,260 validation interactions, and 52,048 test interactions, along with the corresponding protein sequences from SwissProt. The dataset was partitioned to ensure no overlap of proteins between sets (training, validation, and test) and minimized sequence similarity using KaHIP. Additionally, redundancy within each partition was reduced by applying the CD-HIT tool with a 40 % pairwise sequence similarity threshold. This design allows for a more reliable evaluation of models that must capture complex features beyond sequence similarity.

To run the experiments with this gold standard dataset, the internal CV process was applied using only the provided training partition. In this step, the training partition was further split into multiple internal folds to optimize the model and select the best set of hyperparameters. Once the best model was selected through internal CV, its performance was evaluated using the external validation partition provided by the gold standard dataset. The test partition from the gold standard dataset was not employed in this study, as the goal was not to finalize model selection but to evaluate the effectiveness of the metric proposed in this work.

### 3.4 Basic evaluation metrics

In this study, for a given set of binary predictions, where a 2 × 2 confusion matrix can be computed, we derive the Matthews Correlation Coefficient (MCC), which gives a metric to measure how much the model is far from random guessing even in unbalanced datasets. It is defined as follows:

$$\text{MCC}=\frac{\text{TP}*\text{TN }-\text{ FP}*\text{FN}}{\sqrt{\left(\text{TP }+\text{ FP}\right)*\left(\text{TP }+\text{ FN}\right)*\left(\text{TN }+\text{ FP}\right)*\left(\text{TN }+\text{ FN}\right)}}$$

where TP, TN, FP and FN represent true positive, true negative, false positive, false negative counts, respectively.

### 3.5 Per-protein utility metric

**Figure 1:**
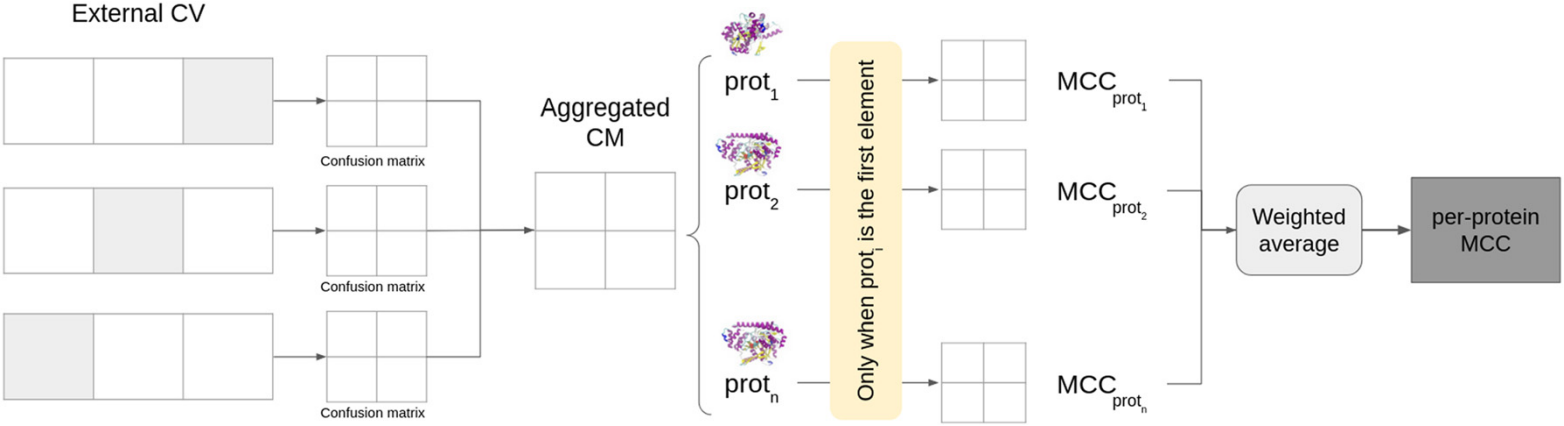
Steps to compute the per-protein MCC.

To evaluate the effect per-protein imbalance in performance metrics, here we propose to evaluate MCC at protein level during validation (Figure 1). First, all folds of the external CV are aggregated into a single confusion matrix. Then, for each protein, we considered only the interactions involving it and computed the MCC on the corresponding confusion submatrix. Then, we computed an average over all proteins, weighted by the number of interactions in which such protein appears in the validation set, to obtain the final performance measures adjusted by protein, that is, the per-protein MCC (pp_MCC). If a given protein does not have true positive interactions or does not have true negative interactions in the test set, the MCC is not computed (zero division, since recall or specificity has zero denominator) and this protein is skipped.

We aimed at providing a kind of utility metric, useful in real scenarios, where, for example, multiple hypothetical ligands are searched for a “reference” protein. In such scenario, we want to get the performance for each protein, for example this “reference” protein. A model that only relies on the learned positivity of the first protein, such as the PPIIBM *singleItemMode* baseline model (see 3.3 in our previous work [21]) will predict all true or all false for the same protein, that is, random guesses (MCC = 0).

Finally, we have chosen the MCC metric as the base for pp_MCC, since it is a popular metric in similar PPI studies, but other metrics (such as F1, AUC, Balanced Accuracy, Youden Index, etc.) could be used and are easy to calculate inside the proposed framework.

## 4 Results and discussion

**Figure 2:**
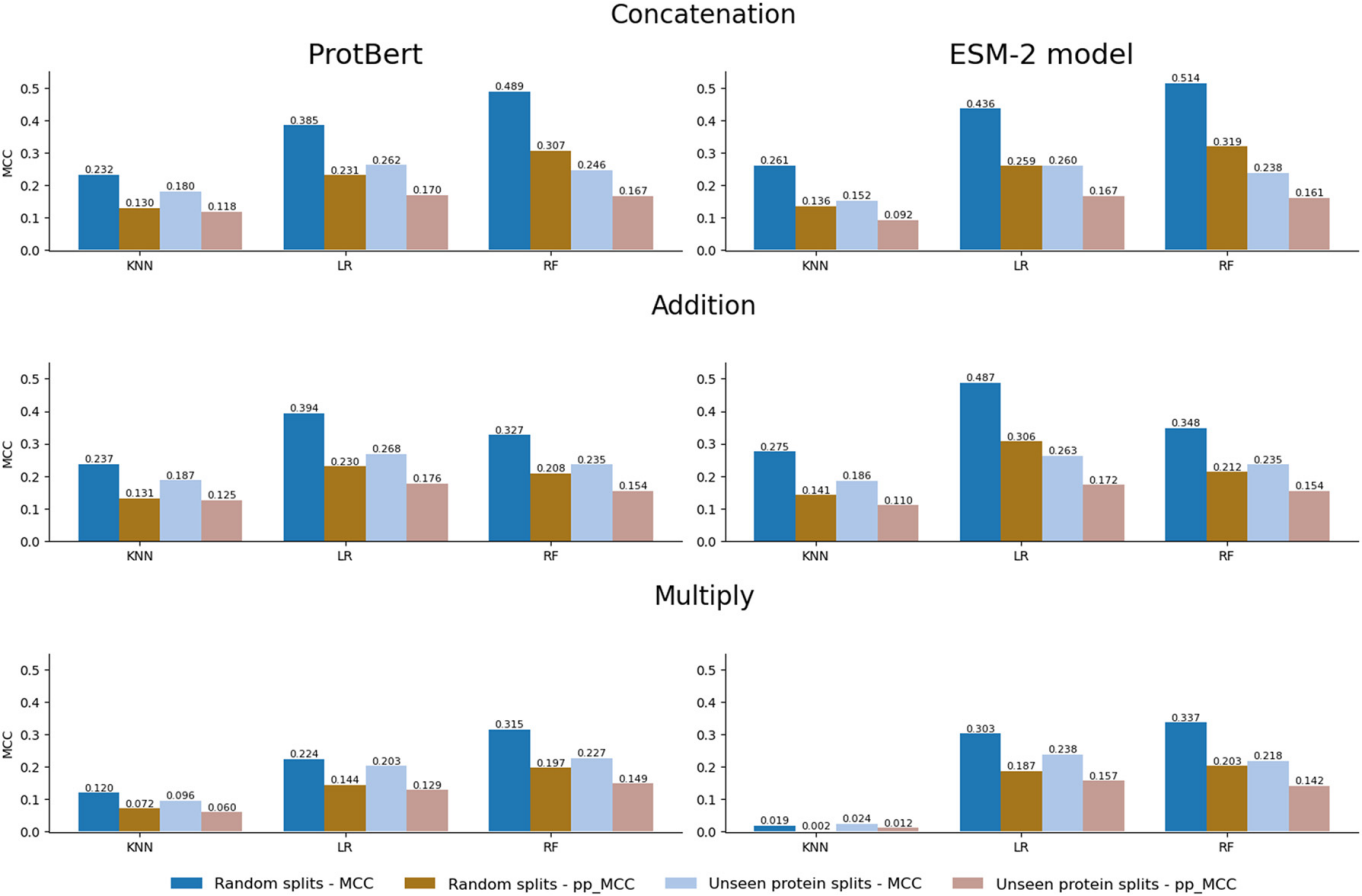
Comparison between the random splits (dark columns) and the unseen protein splits (soft columns) settings. Results are provided by combination (concatenation, addition, or multiply) and embedding (ProtBert or ESM-2 model).

In general terms in the random splits setting, as we can see in Figure 2 (two left columns), we observe a decrease when comparing pp_MCC with raw MCC, evidencing that our metric robustly drops the raw MCC. However, none of the models reach a pp_MCC of 0, meaning that models seem to not only exploit the positivity bias present in the dataset. In this sense, not all models show the same performance. RF models tend to achieve superior performance compared to KNN and LR models in most combinations (embedding and combination) and metrics. Furthermore, the combination of concatenation with the RF model using the pretrained ESM-2 model produced the highest performance (MCC = 0.514). This combination was also the best in the pp_MCC metric (0.319).

Regarding embeddings, ESM-2 seems to have a slightly better performance compared to ProtBert, with the exception of the multiplication combinations in the KNN model. The average raw MCC for ProtBert is 0.303 compared to 0.331 for ESM-2, and for pp_MCC, the average is 0.183 for ProtBert and 0.196 for ESM-2. We have observed that, although the improvement by using ESM-2 is small, it is consistent, and thus embeddings seem to have influence in the final downstream performance.

We also wanted to evaluate the performance of the models in a more challenging setting, where the proteins present in the interactions of the validation split are not used for training, as explained before. As it can be observed in Figure 2, making unseen protein splits leads to worse performance compared to the previous random splits. We observe that, in general, the MCC and pp_MCC values for unseen protein splits are lower than for random splits, which is in line with the published literature stating that random splits allow the models to learn from the same protein, leading to overoptimistic performance. Interestingly the estimated performance obtained by using pp_MCC in the random split is very similar to the raw performance obtained in the unseen proteins split (second and third columns in Figure 2).

Moreover, in the random splits setting, we observe high differences in MCC and pp_MCC values, in comparison with the unseen protein splits setting, where the differences between both metrics are more moderate, suggesting more consistent performance and a more realistic evaluation of the model using the more challenging unseen protein split.

**Figure 3:**
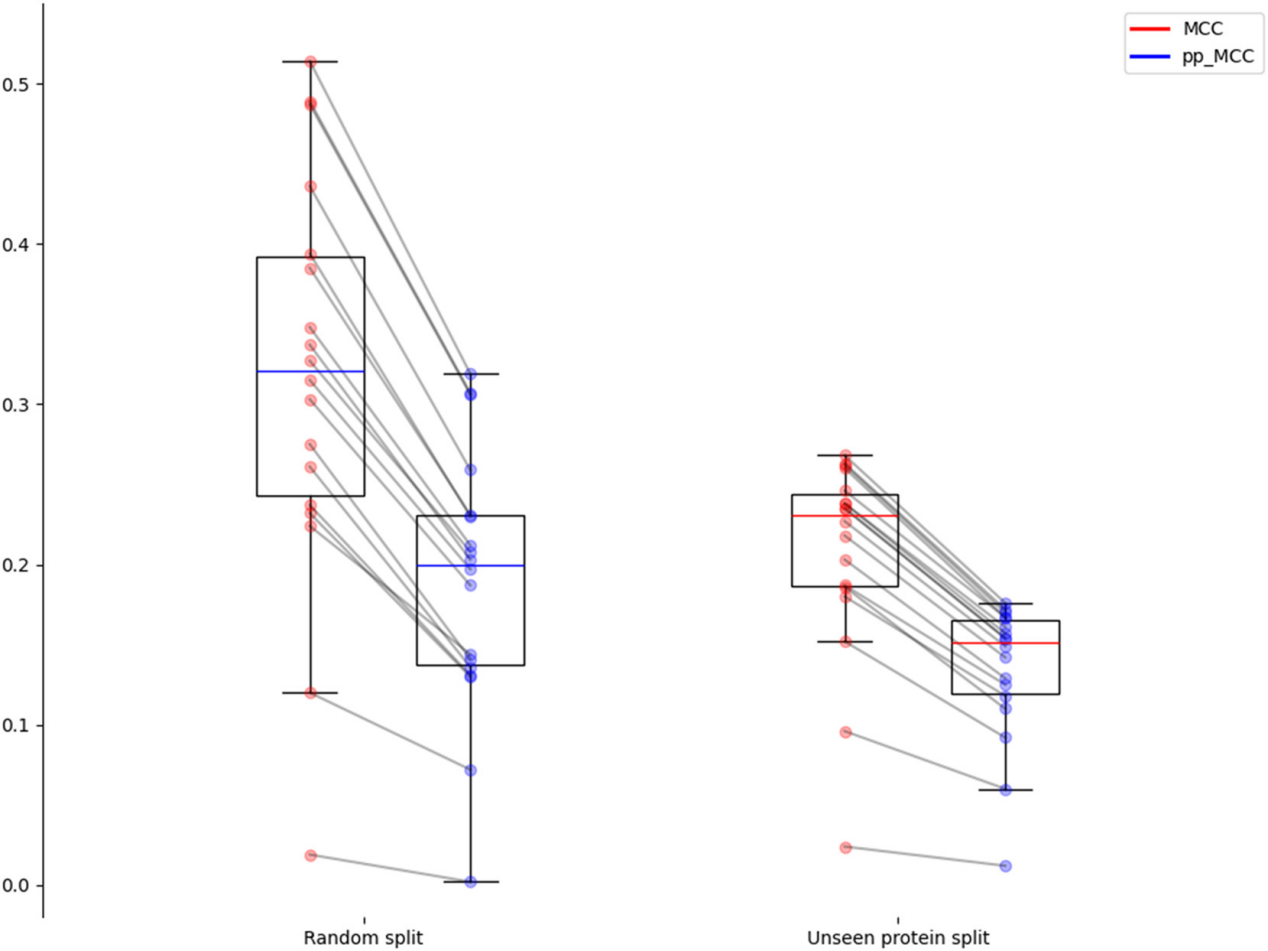
Distribution of MCC and pp_MCC for the random splits and unseen protein splits settings.

Figure 3 shows how both the split strategy and the use of the pp_MCC affect the obtained median performance. Starting from median values slightly above 0.3 in random splits with raw MCC, lowering to values around 0.2 when using protein split or pp_MCC, and reaching floor values around 0.15 if both strategies are combined. Figure 3 also illustrates these two effects: lower dispersion in unseen protein splits and closer MCC and pp_MCC. The differences between MCC and pp_MCC are also statistically significant (*p*-values <0.01 in a paired *t*-test in both settings).

All these findings highlight the importance of considering bias in protein interaction databases and adapting evaluation strategies to obtain more robust and generalizable models, as traditional evaluations (i.e. non-adjusted metrics on random splits) tend to overestimate performance due to the per-protein unbalanced distribution of interactions.

**Figure 4:**
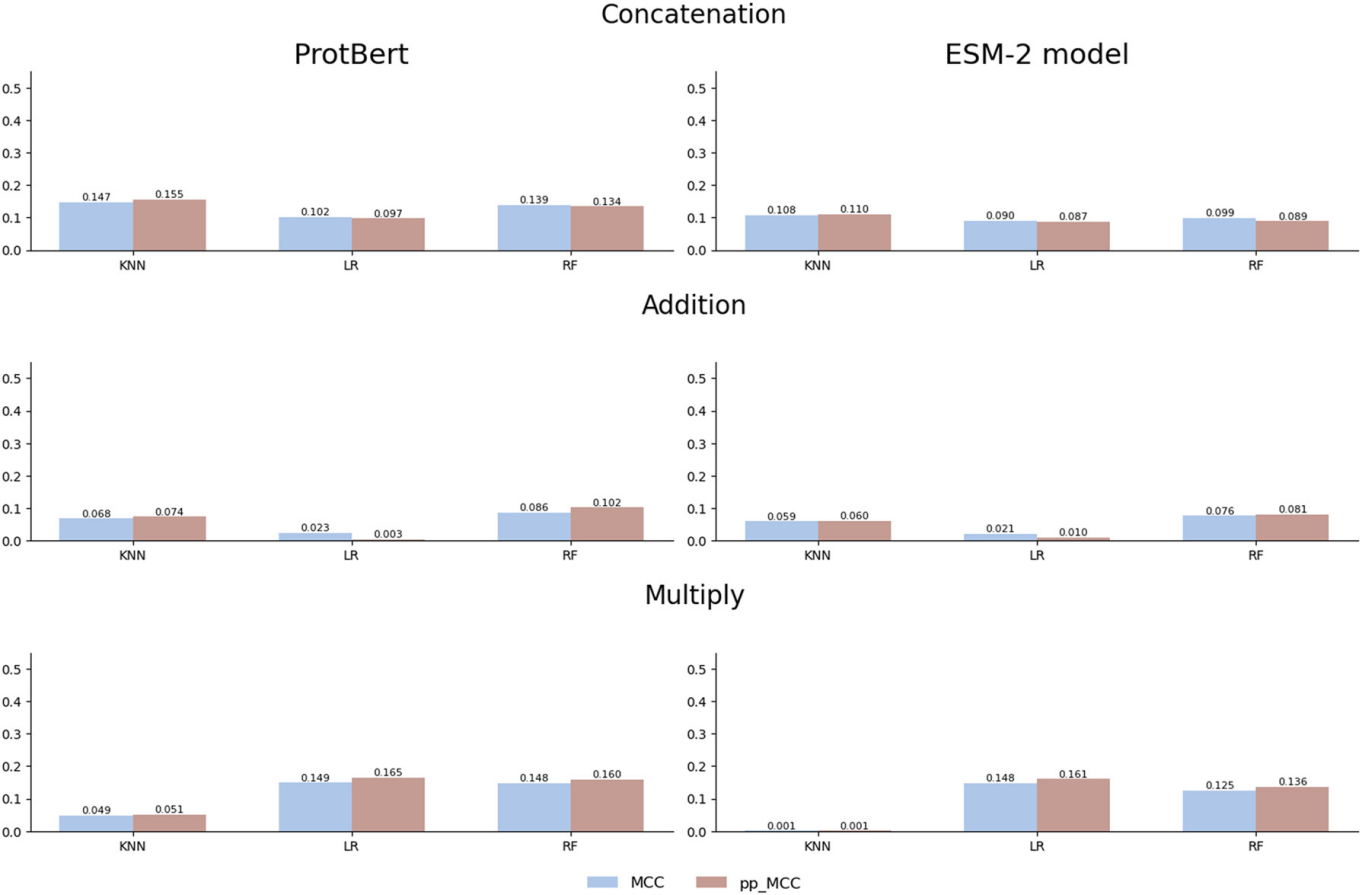
Comparison of MCC and pp_MCC across different models and combination strategies with ProtBert and ESM-2 embeddings on the gold standard dataset.

When a well-constructed dataset is used, such as the gold standard dataset, the values of the raw MCC metric do not present the overestimations (Figure 4). This dataset has been carefully designed by the authors by minimizing similarities between proteins and removing redundancies, which prevents the common biases that artificially inflate model performance. In this context, the proposed pp_MCC metric demonstrates that its utility can also complement traditional metrics, as it reflects the same performance when the dataset is well-constructed.

**Figure 5:**
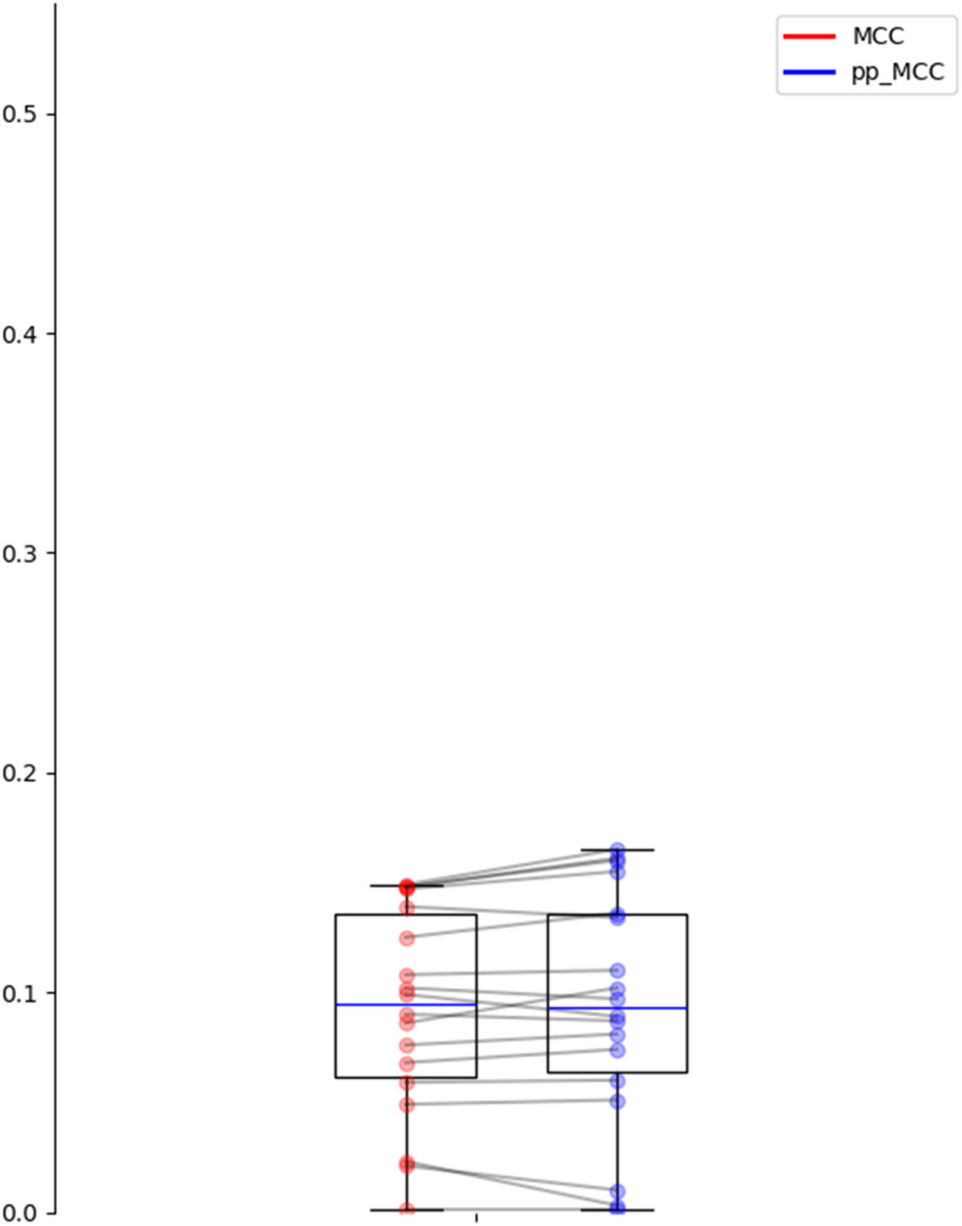
Distribution of MCC and pp_MCC on the gold standard dataset.

Furthermore, when analyzing the distribution of the MCC and pp_MCC metrics, as illustrated in Figure 5, pp_MCC consistently shows similar values compared to MCC, even in this controlled scenario. This highlights its robustness as a metric that adjusts model performance estimates, providing a more realistic perspective. While the primary objective of pp_MCC is to detect biases in poorly constructed datasets, these results confirm that its application is also suitable for validating models in contexts where the data has been carefully curated.

The pp_MCC metric is not only effective in detecting biases in datasets but also ensures that model performance is not artificially inflated, offering a more reliable and generalizable evaluation in any scenario.

## 5 Conclusions

The pp_MCC metric proposed in this work offers a pathway to improve the reliability and interpretation of results in PPI prediction, promoting the development of more effective models. Here we have taken into account and addressed a specific bias in the PPI datasets related to the per-protein unbalanced positivity. As we expected, we obtained generally lower performance estimated after this methodological adjustment, showing their usefulness and highlighting the importance of considering bias in PPI databases and adapting evaluation strategies to obtain more robust and generalizable models, in line with other studies pointing in the same direction [17], 18], 29], 32].

We tested the pp_MCC in two splitting strategies. A random split and a more challenging unseen protein split, which prevents any interaction of the same protein from being seen in the training set, that also could mitigate the bias of identifying the protein and exploiting its positivity, but also hinders learning the biological manner in which that protein interacts with others. In this sense, since the pp_MCC aims to eliminate the effect of bias even in a random split, it allows to still train with other interactions of the to-be-predicted proteins, which could be interesting for some studies that are more protein-centric. For example, we could be interested in studying potential novel interactions of a given protein under study while allowing us to use models that were trained with other interactions of the same protein.

The adjusted performance values were still above random guess (pp_MCC > 0), meaning that models seem to capture real biological information during training. However, the presence of more biases and data leakages exploited by the models could not be absolutely discarded, which warrants further investigation. For example, datasets containing repeated or similar sequences, will produce very similar embeddings. If the positivity of repeated or similar proteins remains unbalanced and in the same direction of the to-be-predicted proteins, the bias would persist. In fact, similar proteins have a tendency to share their ligand networks. Therefore, if we want to evaluate the power of ML primary sequences in 1D space methods based on features other than high overall protein similarity, we should also exclude from the evaluation set the proteins sharing similarity with those used in the training set. This could be done using tools such as CD-HIT [35]. Building on this, we further tested the pp_MCC metric on a rigorously curated gold standard dataset that employed CD-HIT to remove sequence redundancy and minimize similarity between training, validation, and test sets. The results confirmed that both MCC and pp_MCC metrics displayed significantly lower values under these stringent conditions, highlighting the inherent limitations of models trained solely on primary sequence data. Nevertheless, the pp_MCC metric demonstrated its robustness by avoiding overestimated performance, even in the absence of detectable bias. This reinforces its value as a reliable evaluation tool, particularly on datasets that were not carefully split, where a discrepancy between raw metrics and the pp_MCC could indicate the presence of biases in the split. Our results underscore the importance of adopting rigorous data preprocessing and robust metrics to ensure unbiased and generalizable evaluations of protein–protein interaction prediction models.
